# Evaluation of a modified protocol that decreases the turnaround time of positive blood cultures

**Published:** 2018-10

**Authors:** Renuka Anegundi, Raghavendra Dheerendra Kulkarni, Satish S Patil, Vidyavathi B Chitharagi, Ganavalli Subramanya Ajantha

**Affiliations:** 1 Department of Microbiology, Kasturba Medical College, Manipal, Karnataka, India; 2 Department of Microbiology, SDM College of Medical Sciences and Hospital, Dharwad, Karnataka, India; 3 Department of Microbiology, SS Institute of Medical Sciences and Research Centre, Davangere, Karnataka, India; 4 Department of Microbiology, JSS Medical College, Mysore, Karnataka, India

**Keywords:** Modified protocol, Automated blood culture, Neonatal septicemia

## Abstract

**Background and Objectives:**

Neonatal septicemia can be rapidly fatal if not treated promptly. A speedy laboratory diagnosis would improve the outcome. The BacT/ALERT 3D system (bioMerieux, Durham, North Carolina) is currently being used for laboratory diagnosis of blood stream infections. In the present study, a modified protocol was employed in which the broth was subcultured into two nutrient broth tubes and these tubes were used for biochemical tests and antimicrobial susceptibility testing to decrease the turnaround time.

**Materials and Methods:**

A prospective study was conducted in the Department of Microbiology, SDM College of Medical Sciences and Hospital, Dharwad from October 2010 to July 2012 after receiving clearance from the institutional ethics committee. Automated blood cultures of 250 neonates admitted to the Neonatal Intensive Care Unit (NICU), clinically diagnosed to have septicemia, were performed using BacT/ALERT 3D. Bottles flagged positive within 72 hours of loading were processed for identification and antibiotic susceptibility testing using a modified protocol. The results were assessed for time saved in reporting in comparison with standard protocol. Student’s t test was used for statistical analysis.

**Results:**

Of the 250 cases studied, 117 cases yielded a positive blood culture giving a yield of 46.8%. The number of cases yielding monomicrobial growth were 73, which were included for further analysis. Of the remaining samples, 133 did not show growth, 11 were polymicrobial while 33 samples were flagged positive after 72 hours. *Candida* spp. grew in 34 cases, Gram negative bacilli grew in 28 cases and Gram positive cocci grew in 11 cases. In four cases, 66 hours were saved, 60 and 54 hours were saved in 18 cases each, 48 hours were saved in 27 cases, and 24 hours were saved in 6 cases. Methicillin resistant *Staphylococcus aureus* and *Klebsiella pneumoniae* were the most common isolates among Gram positive cocci and Gram negative bacilli, respectively, while *C. guilliermondii* was the most common *Candida* isolate. All Gram positive isolates were susceptible to vancomycin and linezolid. Most of the Gram negative isolates were susceptible to imipenem.

**Conclusion:**

This method can be employed in peripheral laboratory settings where there is no complete automation. Modification in processing blood culture can provide speedy identification and sensitivity report in blood stream infections. Time saved in reporting would play a crucial role in improving morbidity and mortality rates in neonatal septicemia.

## INTRODUCTION

Neonatal sepsis is more common in developing countries and is an important cause of morbidity and mortality (1). The World Health Organisation (WHO) estimates more than 4 million neonatal deaths every year worldwide (2). The condition can be rapidly fatal if not treated promptly, aggressively and specifically. Majority of the blood stream infections are found to be monomicrobial (3, 4). Obtaining a pure culture of the suspected pathogen is an essential step to identification and sensitivity testing. For the standard blood culture protocol; blood is collected into BHI broth or Hartley broth and incubated at 37°C. At the end of 12 to 24 hours incubation, the first subculture is made on solid media; preferably on blood agar and MacConkey’s agar. However, in the majority of cases, 48 hour incubation of blood culture broth is required for optimal growth (5, 6). The colonies grow on solid media at the end of 18 to 48 hours incubation. The colonies are processed for identification by Gram staining and relevant biochemical tests. Another 18 to 48 hours of incubation is essential for drug susceptibility by disc diffusion method. This protocol takes a minimum of four days (96 hours) to provide a blood culture and susceptibility report.

In the present study, a modified protocol was employed, in which two nutrient broth tubes were inoculated with the positive blood culture broths which were used for biochemical tests and antimicrobial susceptibility curtailing the step of obtaining growth on agar plates. The purpose of this work was to study the efficacy of a modified protocol which reduced the turnaround time of reporting which is the cornerstone of reducing neonatal mortality.

## MATERIALS AND METHODS

The study was conducted from October 2010 to July 2012 after obtaining permission from the institutional ethical committee in Department of Microbiology, SDM College of Medical Sciences and Hospital, Dharwad. Blood culture was performed in 250 clinically diagnosed cases of neonatal septicemia by automated blood culture system (BacT/ALERT 3D, bioMerieux, Durham, North Carolina). About 0.5 ml or more of blood was collected by venipuncture from peripheral vein under strict aseptic precautions using 70% alcohol and povidone iodine (7). Sample was directly inoculated into a single pediatric aerobic automated blood collection bottle “pediatric” (Yellow, 20 ml, BacT/Alert PF). The bottle was loaded in the BacT/ALERT 3D Microbial Detection System. The modified protocol was employed for the bottles that flagged positive at or before 72 hours. Blood culture bottles which flagged positive after 72 hours were excluded from the study (8, 9). Of the 250 cases studied, cases yielding monomicrobial growth were included for analysis. Blood cultures with recovery of multiple isolates were excluded from the study.

As soon as the blood culture bottle was flagged positive, the time was noted and modified protocol was followed.

Immediately after a bottle flagged positive, it was inverted several times and 0.5 ml broth was drawn using a 2 ml sterile disposable syringe in a biosafety cabinet level II A2 (Kartos International, Noida). One drop of the aspirate was seeded on a blood agar and a MacConkey’s agar plate. One drop from every aspirate was added to two nutrient broth tubes and the tubes were placed in incubator at 37°C. One drop each was put on two glass slides and smears were prepared and heat fixed.

One of the smears was stained with Gram stain. If the Gram staining confirmed monomicrobial infection, the Gram staining report was immediately sent to treating paediatrician. The other smear was preserved for methylene blue staining if Gram staining did not reveal any pathogen. The nutrient broth tubes were incubated for two hours. Turbidity of the other tube was adjusted to 0.5 McFarland and sensitivity was put according to Gram staining result. The second nutrient broth tube was used to setup biochemical and physiological reactions for identification. The colony morphology on solid media, biochemical reactions and sensitivity test results were observed the next day to finalize culture and sensitivity report. When the smear showed growth of yeast, it was subcultured on Sabouraud’s Dextrose agar. Germ tube test was performed from the colonies and the result was communicated to the treating paediatrician. Identification of the yeast was done using sugar assimilation test and growth on Corn meal agar.

If the smear showed more than one type of organism, identification and sensitivity tests were performed by conventional protocol from the blood and MacConkey’s agar plates. The bottles that did not flag positive were confirmed by Gram staining and methylene blue staining to declare as “No Growth”.

### Standard protocol (10).

Blood is collected into BHI broth or Hartley broth and incubated at 37°C. At the end of 12 to 24 hours incubation the first subculture is made on solid media; preferably on blood agar and MacConkey’s agar. If there is no growth, subculture will be done on fourth and seventh day of inoculation of the sample. The colonies grew on solid media at the end of 18 to 48 hours incubation. The colonies were processed for identification by Gram staining and relevant biochemical tests. It takes at least 48 hours in the majority of samples to get optimum growth (5, 6). Final report with antimicrobial susceptibility takes further 48 hours making it a total of 96 hours.

### Modified protocol.

**Fig. 1.**
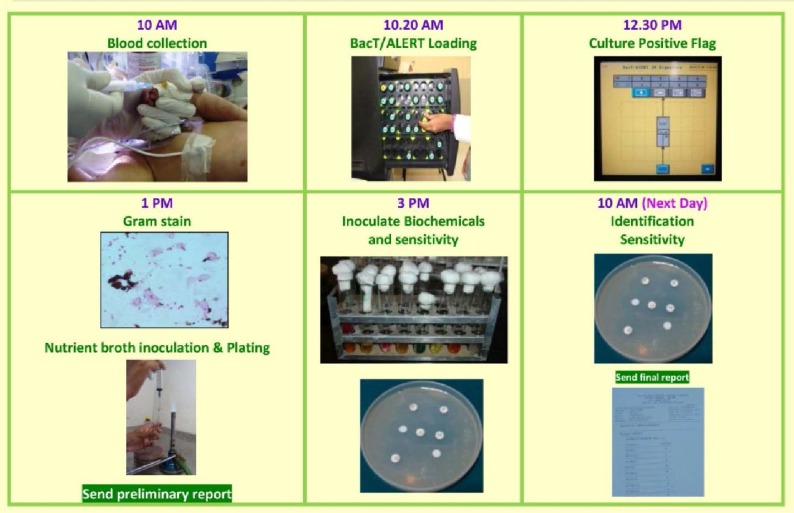
Modified Protocol for blood culture

Collect blood samples directly into BacT/ALERT 3D bottles. If BacT/ALERT flags positive, make a Gram stained film and convey report to ward. Make a subculture from the broth on blood agar and MacConkey’s agar or Sabouraud dextrose agar. If the growth is monomicrobial on Gram staining, inoculate two tubes of nutrient broth with one drop each from the broth in culture bottle. Incubate nutrient broth tubes for 4 to 6 hours. Use one tube for biochemical and physiological reactions. Adjust the turbidity of the other tube to McFarland No. 0.5 and use it for antimicrobial susceptibility testing as per CLSI guidelines (11). Report identification and sensitivity next day morning after careful review of the growth on plates, biochemical reactions and antibiotic sensitivity (Fig. 1).

### Statistics.

Student’s t test was used to calculate the efficacy of modified protocol in comparison with the standard protocol.

## RESULTS

Of the 250 cases studied, 117 yielded positive blood culture of which 73 cases yielding monomicrobial growth and were included for analysis. Of the remaining samples, 133 did not show growth, 11 were polymicrobial while 33 samples flagged positive after 72 hours. Samples showing bacterial growth were 39, *Candida* spp. were yielded by 34 and 11 showed polymicrobial growth accounting for a total of 84 samples giving positive blood culture (33.6%). *Klebsiella pneumoniae* were the predominant bacterial isolates accounting for 28 cases out of 39. A total of 11 samples gave Gram positive cocci of which methicillin resistant *Staphylococcus aureus* were the most common. Among the 34 yeast isolates the most common was *C. guilliermondii* accounting for 10 isolates while *C. albicans* was cultured only from two cases (Table 1). All Gram positive isolates were susceptible to vancomycin and linezolid. Most of the Gram negative isolates (81.5%) were susceptible to imipenem.

**Table 1.** Bacterial and fungal isolates recovered during the study

**Blood culture results (n = 250)**	**Number**
Blood culture positive	117
Monomicrobial growth	73
Bacterial isolates	39
• Gram positive	28
• Gram negative	11
Yeasts	34

### Time to positivity.

With consideration of conventional reporting time of 96 hours; 66 hours were saved in four cases, 60 hours saved in 18 cases, 54 hours in 18 cases, 48 hours in 27 cases, and 24 hours were saved in 6 cases (Table 2). The median time taken by the 73 samples to flag positive was 18 hours. The difference between the median times taken for reporting by BacT/ALERT3D (42 hours) and conventional method (96 hours) by Student’s t test yields a high, significant difference of p< .0011, (t = 6.95, df = 4). This indicates that there is a net saving of 54 hours (96 minus 42 hours) (Table 2).

**Table 2.** Time to positivity of 73 isolates and comparison of time saved against conventional technique

**No. of Samples (n = 73)**	**Flagged Positive**	**Final report sent with antimicrobial sensitivity**		**Time Saved**

		**BacT/ALERT3D**	**Standard protocol**	
4	6 hours	30 hours	96 hours	66 hours
18	12 hours	36 hours	96 hours	60 hours
18	18 hours	42 hours	96 hours	54 hours
27	24 hours	48 hours	96 hours	48 hours
6	48 hours	72 hours	96 hours	24 hours
18 Hours		42 Hours	96 Hours	54 Hours
Median		Median	Median	Median

## DISCUSSION

Neonatal sepsis is a life threatening emergency and delay in treatment may lead to fatality (12). Infections are the single most prevalent cause of neonatal death contributing for 35% of neonatal deaths (13). In the present study, 73 cases were found to have monomicrobial and 11 were polymicrobial infections. Since these positive blood cultures with monomicrobial infection are, in reality, pure broth cultures or bacterial suspensions, they can directly be used for identification and antimicrobiol susceptibility testing. This concept was used to design the modified protocol.

In our study, a total of 36 samples flagged positive within 18 hours followed by 27 samples within 24 hours. A 2001 study by Kumar et al. showed highest positivity within 12 to 24 hours followed by 24 to 36 hours (8). Similar results were reported by Guerti et al., showing 155 isolates positive in 12 to 24 hours and 141 isolates in 24 to 48 hours (14).

Neonatal blood stream infections carry higher bacterial load compared to adults (10). The greater the initial bacterial load, the lesser the time taken to flag positivity. Thus, high bacterial load samples give early positivity and low bacterial load samples may show delayed flag positivity. High initial bacterial load is associated with poor prognosis (10).

By using the modified protocol, we saved maximum 66 hours to minimum 24 hours in dispatching the report in comparison to the conventional blood culture technique. The blood culture broth was directly used for biochemical testing and antimicrobial susceptibility test without the use of growth from culture plates. Oopta et al. in 2015 showed importance and use of positive blood culture broth pellet which was subjected to various automated systems for identification and antimicrobial susceptibility test (15). A study by Machen et al. in 2014 found the average time to identification and antimicrobial susceptibility testing to be 11.4 hours, using Lysis-Filtration method for both VITEK MS and VITEKH2 compared to 56.3 hours by using conventional methods (16). Adoption of this protocol in the day to day practice at our institute is possible as sufficient laboratory staff is available around the clock here. The protocol followed would be useful mainly for the laboratories where automated Vitek systems are not available.

The limitations of this study could be lack of use of the pellet of positively beeped blood culture broths as was used by some workers (15, 17). However, an additional step of pelleting might lead to contamination. Antimicrobial susceptibility testing done from direct broth is also suggested (18), but it is necessary to bring the turbidity to McFarland’s 0.5 to achieve standard inoculums which may not be the case in a bottle flagging positive. We took the approach of correlating the colonies on the agar plates of broth subcultures, biochemical test results and purity of lawn on sensitivity plate. Putting up a sensitivity test in parallel from the colonies could have been used for more accurate evaluation. This protocol is not useful in polymicrobial blood stream infections.

## CONCLUSION

As the nutrient broth was used directly for culture, biochemical tests and antimicrobial susceptibility testing, there was significant decrease in the turnaround time. Using the modified protocol, the automated blood culture systems can be used to provide speedy identification and susceptibility report in blood stream infections. A complete culture and susceptibility report can be provided even within 24 hours of receipt of the sample. Precise identification of the causative agent and time saved in such cases would play a crucial role in reducing the morbidity and mortality in the neonates with septicemia. Therefore, time saved would certainly play a crucial role in these cases and would reduce the morbidity and mortality.
